# Endotoxemia and mortality prediction in ICU and other settings: underlying risk and co-detection of gram negative bacteremia are confounders

**DOI:** 10.1186/cc11462

**Published:** 2012-08-07

**Authors:** James C Hurley, Bertrand Guidet, Georges Offenstadt, Eric Maury

**Affiliations:** 1 Rural Health Academic Center, Melbourne Medical School, 'Dunvegan' 806 Mair St., University of Melbourne, Ballarat, Victoria 3350, Australia; 2 Division of Internal Medicine, Ballarat Health Services, 101 Drummond St., N, Ballarat, 3350, Victoria, Australia; 3 Réanimation médicale, Assistance Publique - Hôpitaux de Paris, Hôpital Saint-Antoine, 184 rue du Faubourg Saint Antoine, Paris, F-75012, France; 4 UPMC Université Paris 06, 4 Place Jussieu, Paris, 75005, France; 5 Inserm, Unité de Recherche en Épidémiologie Systèmes d'Information et Modélisation (U707), Paris, F-75012, France

## Abstract

**Introduction:**

The interdependence between endotoxemia, gram negative (GN) bacteremia and mortality has been extensively studied. Underlying patient risk and GN bacteremia types are possible confounders of the relationship.

**Methods:**

Published studies with ≥10 patients in either ICU or non-ICU settings, endotoxemia detection by limulus assay, reporting mortality proportions and ≥1 GN bacteremia were included. Summary odds ratios (OR) for mortality were derived across all studies by meta-analysis for the following contrasts: sub-groups with either endotoxemia (group three), GN bacteremia (group two) or both (group one) each versus the group with neither detected (group four; reference group). The mortality proportion for group four is the proxy measure of study level risk within L'Abbé plots.

**Results:**

Thirty-five studies were found. Among nine studies in an ICU setting, the OR for mortality was borderline (OR <2) or non-significantly increased for groups two (GN bacteremia alone) and three (endotoxemia alone) and patient group one (GN bacteremia and endotoxemia co-detected) each versus patient group four (neither endotoxemia nor GN bacteremia detected). The ORs were markedly higher for group one versus group four (OR 6.9; 95% confidence interval (CI), 4.4 -to 11.0 when derived from non-ICU studies. The distributions of *Pseudomonas aeruginosa *and *Escherichia coli *bacteremias among groups one versus two are significantly unequal.

**Conclusions:**

The co-detection of GN bacteremia and endotoxemia is predictive of increased mortality risk versus the detection of neither but only in studies undertaken in a non-ICU setting. Variation in GN bacteremia species types and underlying risk are likely unrecognized confounders in the individual studies.

## Introduction

The prognostic value of endotoxemia detection has been studied in more than forty studies [1-41]. Conflicting conclusions became apparent from the earliest studies undertaken [24,25,41].

The prognostic value remains unresolved despite 17 large studies including more than 2,000 patients [11,21,24-27,29-33,35-41]. On the one hand, in six studies endotoxemia was predictive of septicaemia onset or severe illness [11,21,24,38,40] and hospital mortality [11,24,26] among studies of hospitalized patients unrestricted to an ICU setting. On the other hand, 13 studies including eight among patients restricted to ICU settings found the detection of endotoxemia either did not predict organ dysfunction or mortality [25,27,30,31,33,39,41], predicted mortality but not organ dysfunction [35], predicted organ dysfunction but not mortality [29,32,37,38], or predicted mortality only when the level of endotoxemia was combined within a lipo-polysaccharide cytokine score [36]. In only three [11,21,24] of these 17 studies did the mortality difference between the groups positive versus negative for endotoxemia exceed 20 percentage points.

Several additional clinical observations indicate that the inter-relation between endotoxemia, gram negative (GN) bacteremia and outcome is not simple [42-44]. Less than two thirds of patients with GN bacteremia have endotoxemia detected and vice versa [42]. The concordance with GN bacteremia varies with GN bacteremia species type [43]. The structure function activity of lipid-A, the biologically active component of endotoxin differs for different GN bacteria [45]. Furthermore, the impact of underlying risk of death is a key factor in the clinical setting [46,47] but is difficult to investigate in laboratory studies [48-52]. These factors illustrate the 'disconnect' between attempts to study sepsis in animal models versus the clinical setting [50]. The objective here is to evaluate the GN bacteremia species type and underlying patient risk as possible confounding factors of the prognostic value of endotoxemia as detected using the limulus assay in published clinical studies of patients across a broad spectrum of risk.

## Materials and methods

### Data sources

**Figure 1:**
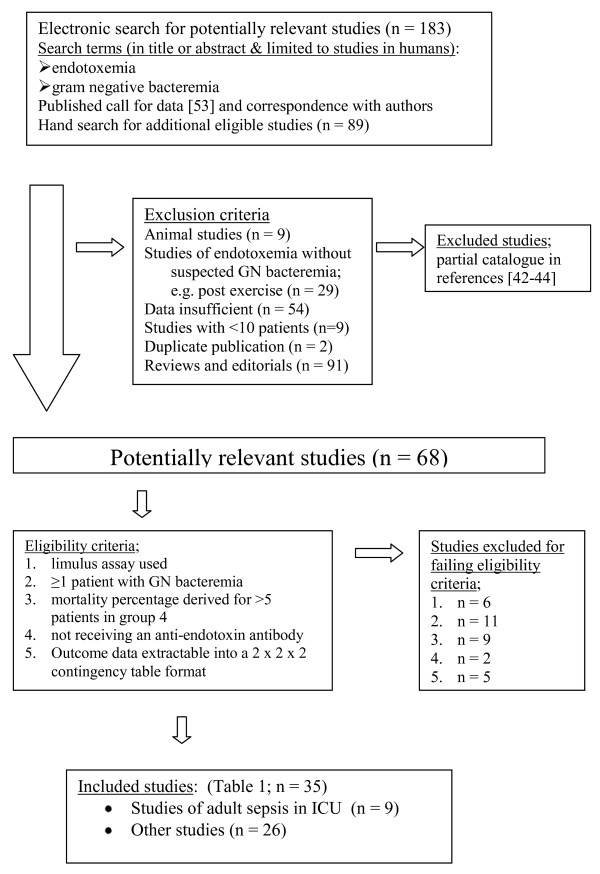
**Flow diagram of study selection within the meta-analysis**.

A computerized search of PUBMED (including Medline) was undertaken using the following key words in the title or abstract; 'endotoxemia', 'limulus' and was restricted to studies in humans. This search was supplemented by a hand-search for studies reporting mortality outcome data in relation to endotoxemia detection and detection of GN bacteremia with blood culture for patient groups at risk of GN bacteremia. This search has been performed repeatedly over two decades [44] up to April 2012 as detailed previously [43,44]. A call for data was published [53] and authors were contacted for additional data to enable inclusion. The flow chart of the literature search strategy and study accrual and disposition is detailed in Figure 1.

### Study selection

The inclusion and exclusion criteria and numbers of studies excluded are detailed in Figure 1. The following inclusion criteria were used; (1) limulus assay used for endotoxemia detection, (2) at least one patient with GN bacteremia, (3) at least ten patients in the study, (4) at least five patients in group four, (5) no anti-endotoxin intervention in use and (6) data was extractable into a 2 × 2 × 2 contingency table format in relation to the co-detection of GN bacteremia and endotoxemia and mortality proportions.

### Data extraction

The patients in all studies were classified into one of four groups as follows: both endotoxemia and GN bacteremia detected (group one), only GN bacteremia detected (group two), only endotoxemia detected (group three), and neither detected (group four). For each of the four groups, the proportion of deaths and denominator data were extracted as a 2 × 2 × 2 contingency table format. Additional data extracted were the type of patient population, whether the study was undertaken in an ICU or another setting and the bacterial species of GN bacteremia isolates.

### Data analysis

There are three objectives of this analysis. Firstly, to determine the prognostic value associated with the detection of GN bacteremia and endotoxemia, each when detected in isolation (groups two and three, respectively) and when co-detected (group one) versus patients for whom neither was detected (group four). This was done by deriving summary odds ratios (OR) and 95% confidence intervals (CIs) using random effects meta-analysis [54] together with an assessment of inter-study statistical heterogeneity using the I^2 ^test for group one, group two and group three each versus group four among the included studies [55]. This was repeated for the sub-groups of studies that included studies of adult patients with sepsis in an ICU setting (defined here as high risk studies) versus studies in other settings (low risk studies). Secondly, to visually compare individual study results using forest and L'Abbé plots [56] the proportion of deaths in groups one, two and three each versus the proportion of deaths in group four as representing the reference group for underlying risk for each study. The line of equality (x = y) is displayed as a visual aid to asses dispersion of the individual study results within each L'Abbé plot. Thirdly, to asses the uniformity in distributions of key GN bacteremia species type among group one and two among those studies for which this information was available. Ethics approval was not required for this study.

## Results

Thirty-five studies [1-35] were found of which 14 studies were supplemented with data provided by personal communication (Table 1). The survival outcome was reported for a total of 3,235 patients among these 35 studies of which 432 (13%), 272 (8%), 1,091 (34%) and 1,440 (44%) were in groups one to four, respectively. Patient inclusion for nine of the studies was based on various criteria for sepsis in adult patients in an ICU setting. A total of 26 of the 35 studies were published within the 1980s and 1990s. The largest study [35] provided mortality data stratified in relation to endotoxemia detection at two breakpoints.

**Table 1:** The included studies.

Study [reference]	Patient population and location	Numbers of patients with fatal outcome/Number tested			
	
		Group 1	Group 2	Group 3	Group 4
		
		Gnb+	Gnb+	Gnb-	Gnb-
		Etx+	Etx-	Etx+	Etx-
**Pre-defined patient groups: Pediatric**					
				
Ahmed *et al. *2004 [1]	Diarrheal illness, hospitalized	3/9	1/1	1/12	1/19
Casey *et al. *1992 [2]	Pediatric cardiac surgery, ICU	0/1	ND	0/15	0/8
Cooperstock, 1985 [3]	Suspected sepsis, hospitalized	0/10	0/1	0/6	0/26
Klein *et al. *1988 [4]	Malnourished children, hospitalized	ND	0/1	0/7	0/8
Shenep *et al. *1988 [5]	Suspected sepsis, hospitalized	2/9	0/1	1/3	0/13
				
**Pre-defined patient groups: Surgery and peri-procedural**					
				
Bailey *et al. *1976 [6]	Obstructive jaundice, hospitalized	1/1	ND	0/12	0/11
Berger *et al. *1995 [7]^a ^	Post-colonoscopy, unspecified location	0/1	ND	0/20	0/11
Foulis *et al. *1982 [8]^f^	Acute pancreatitis, hospitalized	1/1	ND	2/12	1/13
Lau *et al. *1996 [9]^a^	Acute cholangitis, hospitalized	1/8	0/3	0/20	0/9
Lumsden *et al. *1989 [10]	Percutaneous biliary drainage, hospitalized	0/1	ND	0/13	0/7
				
**Pre-defined patient groups: Specified infections**					
				
Brandtzaeg *et al. *1989 [11]^a^	Meningococcal disease, hospitalized	9/24	0/11	ND	0/7
Brandtzaeg *et al. *1996 [12]^a^	Meningococcal disease, hospitalized	21/40	0/19	1/4	3/26
Butler *et al. *1973 [13]^b^	Plague, hospitalized	1/2	ND	0/7	0/1
Butler *et al. *1976 [14]^b^	Plague, hospitalized	0/3	0/2	ND	0/5
Magliulo *et al. *1976 [15]^c^	Salmonellosis, hospitalized	0/1	ND	0/9	0/12
Magliulo *et al. *1976 [15]^c, d^	Typhoid, hospitalized	0/8	0/4	0/1	0/1
Adinolfi *et al. *1987 [16]^d^	Typhoid, hospitalized	0/7	0/7	0/2	0/5
Suyasa *et al. *1995 [17]^d^	Typhoid, hospitalized	1/4	1/6	ND	0/9
				
**Pre-defined patient groups: Oncology and transplant patients**					
				
Bion *et al. *1994 [18]	Elective liver transplantation, ICU	0/1	0/1	1/31	4/19
Engervall *et al. *1997 [19]^a^	Febrile, oncology (80% neutropenic), hospitalized	1/2	0/4	0/4	0/14
Hynninen *et al. *1995 [20]	Febrile, oncology (43% neutropenic), hospitalized	0/3	1/24	ND	10/96
Yoshida *et al. *1994 [21]^a^	Febrile, oncology (63% neutropenic), hospitalized	14/21	1/9	11/35	8/71
				
**Pre-defined patient groups: Other**					
				
Byl *et al. *2001 [22]^a^	Suspected sepsis, hospitalized	2/8	0/4	0/3	1/12
Giamarellos *et al. *1999 [23]^a^	Acute pyelonephritis, hospitalized	1/3	0/9	0/4	0/9
Levin *et al. *1972 [24]	Suspected sepsis, hospitalized	14/20	4/14	7/16	27/168
Stumacher *et al. *1973 [25]	Suspected bacteremia, hospitalized	6/28	15/37	2/18	4/34
Van Langervelde *et al. *2000 [26]^a^	Febrile hospital admissions, hospitalized	7/24	0/24	9/76	16/324
				
**Adult patients in ICU with sepsis as contemporaneously defined**					
				
Bates *et al. *1998 [27]^a^	Sepsis syndrome, multi-center cohort, hospitalized	5/10	12/39	42/109	59/198
Billard *et al. *1994 [28]	Septic shock, ICU	5/6	ND	1/4	4/8
Danner *et al. *1991 [29]^a^	Clinically defined septic shock, ICU	4/11	0/8	8/32	12/49
Dofferhoff *et al. *1992 [30]	Clinically defined severe sepsis, ICU	2/4	0/2	0/6	1/6
Goldie *et al. *1995 [31]^a^	Sepsis syndrome, ICU	5/9	2/3	36/83	18/38
Guidet *et al. *1994 [32]^a^	Sepsis syndrome, ICU	13/24	4/9	14/20	18/40
Strutz *et al. *1999 [33]	Sepsis syndrome, ICU	1/5	3/5	5/8	4/10
Wortel *et al. *1992 [34]^e^	Sepsis syndrome, multi-center cohort, ICU	6/8	4/5	2/3	13/25
Opal *et al. *1999 [35] (Low; >20 pg/ml) ^a, f^	Sepsis syndrome, multi-center cohort, ICU	12/51	7/21	78/255	32/128
Opal *et al. *1999 [35] (High; >660 pg/ml) ^a, f^	Sepsis syndrome, multi-center cohort, ICU	21/63		84/241	

^a^Data for these studies [7,9,11,12,19,21-23,26,27,29,31,32,35] provided by personal communication; ^b^two studies of plague were aggregated for this analysis [13,14]; ^c^this study stratified into two sub-studies of typhoid and salmonellosis [15]; ^d^three small studies of typhoid were aggregated for this analysis [15-17]; ^e^only the patients randomized to receive placebo from this study [34]. ^f^the mortality proportion data for patients with endotoxemia detected from this study [35] has been stratified at two breakpoints, 20 and 660 pg/ml. Etx, endotoxemia; GNB, Gram negative bacteremia; ND, no data.

The species types of GN bacteremia isolates were identified for 31 of the 35 studies [See Additional file 1]. Among the mono-microbial GN bacteremias, there were 174 (26%), 134 (22%), 74 (12%), and 94 (15%) bacteremias with *Escherichia coli*, Enterobacteriaceae other than *E. coli *(for example, *Klebsiella species*, *Enterobacter species*), *Pseudomonas aeruginosa*, and *Neisseria meningitides*, respectively. After excluding studies restricted to specified infections, there were 497 GN bacteremias with species type known among which there was an uneven distribution of *E. coli *versus *P. aeruginosa *identified among the GN bacteremias of group one versus group two; *E. coli *was less common in group one than in group two (92 of 303 (30%) versus 82 of 194 (42%), *P *= 0.007; chi-square test). By contrast, *P. aeruginosa *was more common in group one than in group two (53 of 303 (17%) versus 23 of 194 (12%), *P *= 0.09; chi-square test). This reciprocal mal-distribution was also apparent among the nine studies of adults with sepsis in an ICU setting (data not shown).

### Meta-analysis

The summary OR for death for group one, group two and group three each, respectively, versus group four, are presented in Table 2 for all studies and for the subsets of ICU and non-ICU studies. With only the nine studies undertaken in an ICU considered, the summary ORs were all either not significant or borderline (OR <2).

**Table 2:** Summary odds ratios derived from studies stratified by underlying mortality risk.

Strata of studies		Groups 1 (Endotoxemia and GN bacteremia detected) versus groups 4 (neither detected)		Groups 2 (GN bacteremia alone detected) versus groups 4 (neither detected)		Groups 3 (Endotoxemia alone detected) versus groups 4 (neither detected)	
		
	Number of studies	Odds ratio (95% CI)	I^2 a^ %	Odds ratio (95% CI)	I^2 a^ %	Odds ratio (95% CI)	I^2 a^ %
ICU studies	9	1.5	0%	1.2	0%	1.4	0%
		1.01 to 2.1		0.74 to 2.0		1.09 to 1.8	
							
non-ICU studies	26	6.9	0%	1.5	0%	1.9	0%
		4.4 to 11.0		0.78 to 2.7		1.2 to 2.9	

All studies	35	3.1	42%	1.3	0%	1.5	0%
		2.0 to 4.8		0.89 to 1.9		1.2 to 1.8	

^a^I^2 ^is the measure of heterogeneity in odds ratio between studies with 0% equivalent to no heterogeneity. CI, confidence interval; GN, gram negative.

With the 26 studies undertaken outside of an ICU setting considered, the summary OR for groups one (co-detection of endotoxemia with GN bacteremia) versus group four was 6.9 (4.4 to 11.0) whereas the summary OR for groups two (GN bacteremia alone) and three (endotoxemia alone) versus group four (neither) were not significant or borderline (OR <2).

With all 35 studies considered together, the calculated heterogeneity was moderate (I^2 ^= 42%) in association with the OR derived for group one (co-detection of endotoxemia with GN bacteremia) versus group four from all studies whereas the calculated heterogeneity in association with all the other ORs was minimal (I^2 ^= 0%).

### L'Abbé and forest plots

**Figure 2:**
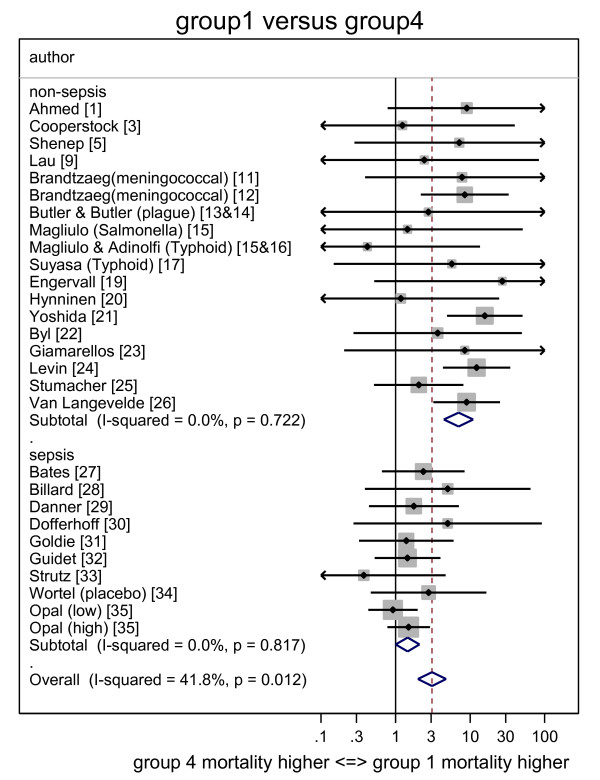
**Forest plot of odds ratios for mortality for Groups 1 (Endotoxemia and GN bacteremia detected) versus groups 4 (neither detected)**. Study specific and summary odds ratios (and 95% confidence intervals) derived from all 35 studies with studies sorted into those conducted in an ICU or non-ICU setting. Arrowheads indicate 95% confidence intervals that extend out of range. GN, gram negative.

**Figure 3:**
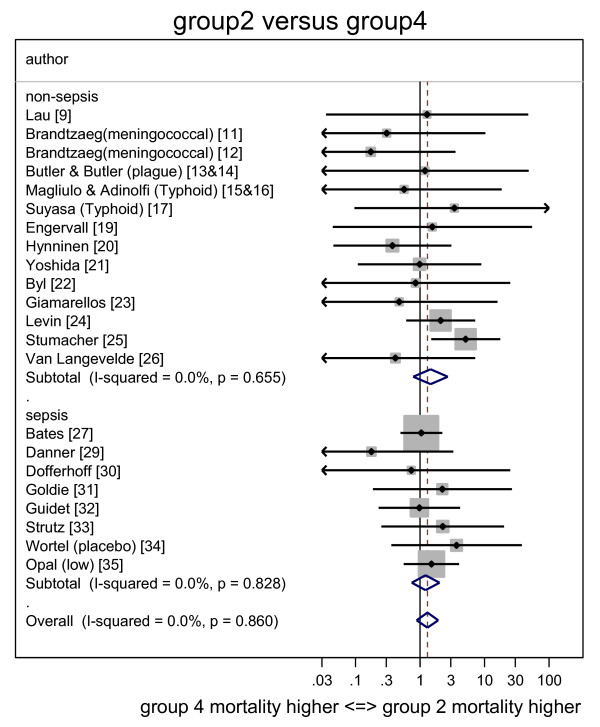
**Forest plot of odds ratios for mortality for Groups 2 (GN bacteremia alone) versus groups 4 (neither detected)**. Study specific and summary odds ratios (and 95% confidence intervals) derived from all 35 studies with studies sorted into those conducted in an ICU or non-ICU setting. Arrowheads indicate 95% confidence intervals that extend out of range. GN, gram negative.

**Figure 4:**
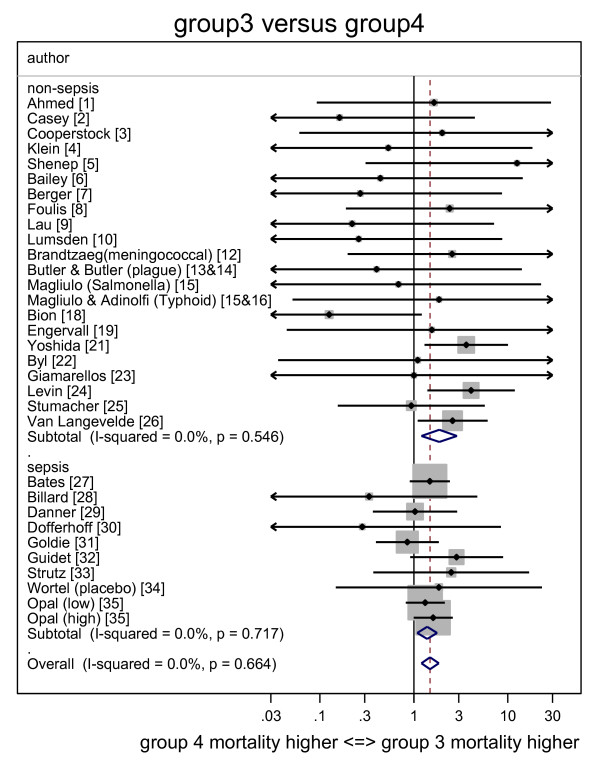
**Forest plot of odds ratios for mortality for Groups 3 (Endotoxemia alone) versus groups 4 (neither detected)**. Study specific and summary odds ratios (and 95% confidence intervals) derived from all 35 studies with studies sorted into those conducted in an ICU or non-ICU setting. Arrowheads indicate 95% confidence intervals that extend out of range.

**Figure 5:**
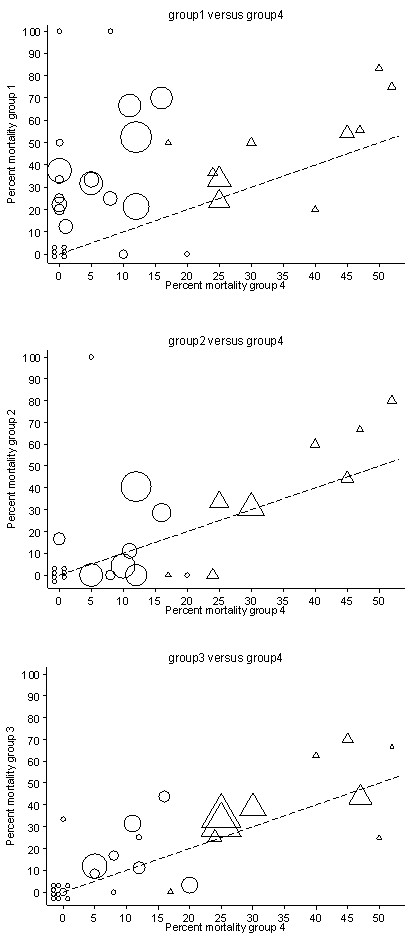
**L'Abbé plots of study specific mortality rates**. Each figure shows mortality rates for studies undertaken in an ICU (triangles) or non-ICU (circles) setting with symbols proportional to group size with the line of no difference (y = x; dotted line) shown for visual reference purposes. Shown are Groups 1 (endotoxemia and GN bacteremia detected) versus groups 4 (neither detected) (Figure 5a - top), Groups 2 (GN bacteremia alone) versus groups 4 (neither detected) (Figure 5b - middle), and Groups 3 (endotoxemia alone) versus groups 4 (neither detected) (Figure 5c - bottom). GN, gram negative.

The individual study ORs together with the summary ORs are presented in forest plots for group one (Figure 2), group two (Figure 3) and group three (Figure 4) all in relation to group four. The corresponding L'Abbé plots are shown in Figure 5a, b, and Figure 5c, respectively.

The group four mortality proportion exceeded 15% for all studies of adult patients with sepsis in an ICU setting (high risk studies) whereas all but one of the other studies (low risk studies) had a group four mortality proportion <15%. The overall dispersion in the mortality proportions away from the line of identity is most apparent in the plot of group one versus group four (Figure 5a), in that for 17 of the 35 studies the mortality proportion in group one was ≥20 percentage points higher in group one versus group four. By contrast, there were only three and five studies in which the mortality proportion for group two or group three, respectively, differed by ≥20 percentage points versus the mortality in group four (Figure 5b and Figure 5c).

When all the included studies here are dichotomized into patient groups positive for endotoxemia (that is, groups one and three aggregated) versus negative (that is, groups two and four aggregated), there were only five studies for which a difference in mortality proportions of ≥20% was apparent in the dichotomy (data not shown) [5,11,12,21,24]. None of these five studies were restricted to populations of ICU patients, four had either an unusually high proportion of bacteremias with *P. aeruginosa *(7 of 30; [21], 7 of 34; [24]) or *N. meningitidis *(35 of 35; [11], 59 of 59; [12]) among the GN bacteremias and the fifth was a pediatric study [5] with 26 patients of which six had bacteremias with either *Hemophilus influenzae, Campylobacter species *or *N. meningitidis*.

## Discussion

This reappraisal of this somewhat dated and disparate literature was undertaken to clarify the following as possible confounding variables toward the prognostic value of endotoxemia: ICU versus non-ICU setting, underlying patient risk, and the species types and distributions of GN bacteremia isolates within the studies. It uses L'Abbe plots to address these questions and adds 20 studies [1-4,6-10,12-19,22,33,35] with 9 received as personal communications, not included in a previous meta-analyses [57].

There are four findings from this analysis. The mortality risk in each of groups one (co-detection of endotoxemia with GN bacteremia), groups two (GN bacteremia alone) and groups three (endotoxemia alone) versus group four (neither) are generally either non-significant, or borderline (OR <2) when derived from only the nine studies in an ICU setting. However, when the 26 non-ICU studies are considered, the risks versus group four are similar to those derived from studies in an ICU setting with the exception of groups one (co-detection of endotoxemia with GN bacteremia), which is markedly increased. Note that these ORs for mortality risk as estimated here are all less than those found in the previous analysis that contained fewer studies [57].

Second, the I^2 ^associated with each summary OR are all 0% with the exception of that associated with the OR for group one versus group four derived from all 35 studies. With this latter exception, this absence of heterogeneity is surprising given the diversity of patient groups, underlying risk, and numbers and settings of these studies that have been conducted and published over a period exceeding three decades.

Third, the underlying patient risk, as reflected in the group four mortality proportion, was higher for studies of patients with sepsis in ICU settings versus studies in other settings (Figure 5a, b, and Figure 5c), as might be expected. However, with the notable exception of the co-detection of group one (endotoxemia with GN bacteremia), the additional risk for each of group two (GN bacteremia alone) and group three (endotoxemia alone) versus group four (neither) is generally similar in the ICU versus the non-ICU setting.

Finally, there are important differences in the prevalence and species types of GN bacteremias among these studies. Of the patients included within the studies of this meta-analysis (not counting GN bacteremias from studies of specified infections or studies in which the species types of GN bacteremia was not stated), 21% had GN bacteremia detected (group one and group two) of which 33% were *E. coli *whereas only 16% were *P. aeruginosa*. However, there are reciprocal differences in the frequency of *P. aeruginosa *versus *E. coli *among the bacteremias of groups one and two. The reciprocal distribution is a consequence of differences in endotoxemia detection rates, being more common for GN bacteremias with *P. aeruginosa *versus *E. coli*. This reciprocal distribution for different GN bacteremia species types is apparent among a broader collection of studies that used the limulus assay [43]. Likewise, among 57 GN bacteremias found in a therapeutic trial in patients with septic shock selected on the basis of a positive detection of endotoxemia using the limulus assay, 15 versus only 12 of the GN bacteremias were *P. aeruginosa *versus *E. coli*, respectively [58].

The relative frequencies of bacteremias with *P. aeruginosa *versus bacteremias with *E. coli *among the studies are of interest for two reasons. The mortality risk for these two common bacteremias differs [59]. For example, in the literature experience over thirty years to 2004, the mortality associated with *P. aeruginosa *bacteremia was typically 32% versus the mortality associated with *E. coli *bacteremia which was typically 19% [60]. The basis for this higher mortality risk is multi-factorial with patient [47,61] and treatment [62,63] factors contributing. Also of interest are the structural differences in lipid-A, the biologically active component of endotoxin (lipopolysaccharide, LPS) [45], specific for *P. aeruginosa *versus *E. coli*. The different lipid-A structures of *P. aeruginosa *versus *E. coli *confer potency differences which are apparent *in vitro *[64,65], and *in vivo *[66]. While the clinical significance of these potency differences is unclear, the resulting unequal distributions of bacteremias among groups one and two uncovered here may confound the apparent relationship between endotoxemia and mortality risk.

## Limitations

This overview is based on a summation of disparate observational studies in an attempt to identify confounders underlying the disparate observations regarding endotoxemia and mortality risk in the literature. This analysis is unable to identify the mechanism for any increased risk. Many relevant patient specific details such as age and co-morbidities for the patients of the four groups in each study were not available. In this analysis, a variable proportion of blood cultures that were classified as negative for GN bacteria would have yielded gram positive bacteremias or fungemias. The detection of endotoxemia in association with blood culture isolates other than GN bacteremias is a commonly reported finding [25,41]. In this respect, the prognostic impact of blood stream infections other than GN bacteremias in relation to the co-detection with endotoxemia has not been addressed here. Moreover, the origin of endotoxemia for patients in group three is uncertain and the possibility of endotoxin originating from other sources, such as gut barrier breakdown, as is presumed to occur for non-septic forms of shock, needs to be considered [67]. Also, it needs to be noted that endotoxemia and GN bacteremia are each either episodic or dynamic [68] phenomena and the criteria for a positive detection of each will have differed among the studies.

The additional mortality associated the co-detection of endotoxemia and GN bacteremia (group one) was less apparent in the studies of patients at high versus low underlying risk (Table 1). This finding here at a group level of analysis resembles other recent findings at an individual patient level of analysis among bacteremias of all species types occurring in an ICU setting [46]. However, this inference requires caution for three reasons.

Firstly, it needs to be clarified as to whether the increased risk is absolute [47] or relative [46].

Second, the proportion of GN bacteremia types that are other than *E. coli *is more variable in studies outside of the ICU setting. In particular, there were five non-ICU studies [5,11,12,21,24] with an unusually high proportion of isolates other than *E. coli *among the GN bacteremias.

Third, it should be cautioned that the L'Abbé plots are useful merely as simple graphical methods to facilitate visual comparisons of the group mortality proportion over the range of underlying risk as found in individual studies within a meta-analysis [56]. The issues underlying the statistical testing for variation in either additional risk or treatment effect in relation to underlying risk are not simple [69,70]. In particular, where linear regression has been used within L'Abbe plots to explore heterogeneity over a range of underlying risk in other contexts, regression to the mean is an important consideration as noted among treatment studies of mild hypertension [71].

In this analysis, group four, which is usually the largest group in each study, serves not only as the reference group for each study in the derivation of the ORs (Table 2) but also as a surrogate marker for the underlying mortality risk within each study (Figures 5a, b, and Figure 5c). However, this proportion will be an imprecise marker in small studies and studies with less restrictive patient selection. As a consequence of this imprecision, proportion estimates from small studies will have reference and comparator groups that by chance will be either more or less extreme than the average and hence, the accompanying comparator or reference group, respectively, will tend to be closer to the mean [72]. This regression to the mean will cause these outlier studies to have a disproportionate impact on the overall relationship with underlying risk and will tend to inflate any deviation of a derived regression relationship with underlying risk away from the null. In the analysis here however, linear regression has not been used and the studies that trended away from the null in the L'Abbe plots were generally neither the smaller studies nor the studies with high event rates among group four.

There were insufficient studies that had used assays other than the limulus assay, or patient groups that had received anti-endotoxin antibodies to include these studies so as to enable a study of these variables by meta-regression. Only seven studies [11-17] were limited to specific GN bacteremia species types. Also, many relevant study level details, such as method of blood culture used and antibiotic therapy protocols were not available. Anti-endotoxin antibodies are detectable in patients with severe sepsis and septic shock and the kinetics of these antibodies over time differs between survivors and non-survivors [73]. However, the impact of these anti-endotoxin antibodies on both the detection of endotoxemia and possibly also on patient outcome within the studies examined here is uncertain. A more detailed examination adjusting for relevant prognostic variables contributing to underlying patient level risk and also the inter-relation between various GN bacteria known to have differing lipid-A structures would require an individual patient data meta-analysis.

With these findings, it is now possible to re-appraise the relationship between endotoxemia detection and mortality risk as observed in the broader literature. With the patient populations dichotomized into endotoxemia positive versus negative, only five [5,11,12,21,24] of the 35 studies included in this analysis found differences in mortality proportions exceeding 20 percentage points (data not shown) and in all five of these studies there was an unusually high proportion of isolates other than *E. coli *(for example, *P. aeruginosa *or *N. meningitidis*) among the GN bacteremias and none of these five studies were restricted to ICU populations. There were a further four large studies in an ICU setting [36-39] that had not been included in this analysis as the mortality proportions were not extractable into a 2 × 2 × 2 contingency table format or used endotoxemia assays other than the limulus assay. None of these four additional studies found differences in mortality proportions exceeding 15 percentage points between endotoxemia positive versus negative patients. While one study had *E. coli *accounting for two of the four GN bacteremias [38], the GN bacteremia species types were not stated in the other three studies. One large non-ICU study did not report mortality as an outcome [40].

While some of the studies were conducted more than thirty years ago, they remain of interest due to the unresolved conflicting conclusions that continue to emerge. These large studies are difficult and expensive to undertake. An understanding of the previous uncertainties would assist in the planning of any further studies to be undertaken.

The extent to which the species type and prevalence of GN bacteremia confounds the relationship between the detection of endotoxemia and outcome and in the evaluation of anti-endotoxin and other adjuvant therapies for sepsis warrants further consideration [74-76].

## Conclusions

The underlying patient risk and the GN bacteremia species types within a study may be unrecognized confounders in the interpretation of the predictive value of endotoxemia. This may not only help to resolve conflicting observations in the clinical literature but may also help to bridge the 'disconnect' with animal models of sepsis. Clarification will help toward defining the exact role of endotoxemia within the pathogenesis of GN sepsis and the evaluation of anti-endotoxin and other adjuvant therapies for sepsis.

## Key messages

• There are conflicting conclusions regarding the prognostic value of endotoxemia detection among more than forty studies in various ICU and non-ICU settings.

• Using the limulus assay for endotoxemia detection, less than two thirds of patients with GN bacteremia have endotoxemia detected and vice versa.

• The mortality risk in association with the detection of endotoxemia or GN bacteremia either alone or together versus the detection of neither is generally either non-significant or borderline (OR <2) when derived from only the nine studies in an ICU setting.

• The co-detection of GN bacteremia and endotoxemia is most predictive of increased mortality risk versus the detection of neither but only in studies undertaken outside of an ICU setting.

• Variation in GN bacteremia species types and underlying risk are likely unrecognized confounders in the individual studies.

## Abbreviations

CI: confidence interval; GN: gram negative; LPS: lipopolysaccharide; ND: no data; OR: odds ratio.

## Competing interests

The authors declare that they have no competing interests. Financial support was provided by The University of Melbourne. The funder had no role in the design, analysis or writing of this study or in its submission for publication.

## Authors' contributions

JCH contributed to the study design, literature search, data analysis, writing and final approval of the manuscript for submission. BG, GO and EM contributed to the literature search, provision of original data and final approval of the submission. All authors read and approved the final manuscript.

## Supplementary Material

Additional file 1 **GN bacteremia types and distributions**. Click here for file
