# Supplementary material for: Endotoxemia and mortality prediction in ICU and other settings: underlying risk and co-detection of gram negative bacteremia are confounders
Source: Crit Care. 2012 Aug 7;16(4):R148. doi: 10.1186/cc11462 (PMC3580737; doi:10.1186/cc11462)
Supplement: Additional file 1 — GN bacteremia types and distributions. [file cc11462-S1.DOC]

**Additional material Table S1: GN bacteremia types and distributions**

|  |  |  | | | | | |
| --- | --- | --- | --- | --- | --- | --- | --- |
| Study [reference] | Patient population & location | E coli | | Pseudomonas | | All | |
|  |  | Group 1 | Group 2 | Group 1 | Group 2 | Group 1 | Group 2 |
|  |  | Etx+ | Etx- | Etx+ | Etx- | Etx+ | Etx- |
|  |  |  |  |  |  |  |  |
| **Pre-defined patient groups: Pediatric** | |  |  |  |  |  |  |
| Ahmed et al 2004 [1] | Diarrhoeal illness, hospitalized | 1 | 0 | 3 | 0 | 9 | 1 |
| Casey et al 1992 [2] | Pediatric cardiac surgery, ICU | 1 | 0 |  | 0 | 1 |  |
| Cooperstock, 1985 [3] | Suspected sepsis, hospitalized | 4 | 0 | 1 | 1 | 10 | 1 |
| Klein et al 1988 [4] | Malnourished children, hospitalized |  |  |  |  |  | 1 |
| Shenep et al 1988 [5] | Suspected sepsis, hospitalized | 0 | 1 | 1 | 0 | 9 | 1 |
| **Pre-defined patient groups: Surgery & peri-procedural** | |  |  |  |  |  |  |
| Bailey et al 1976 [6] | Obstructive jaundice, hospitalized | 1 | 0 | 0 | 0 | 2 |  |
| Berger et al 1995 [7] ^a^ | Post-colonoscopy, unspecified location | 1 | 0 | 0 | 0 | 1 |  |
| Foulis et al 1982 [8] | Acute pancreatitis, hospitalized | 1 | 0 | 0 | 0 | 1 |  |
| Lau et al 1996 [9] ^a^ | Acute cholangitis, hospitalized | 5 | 3 | 0 | 0 | 8 | 3 |
| Lumsden et al 1989 [10] | Percutaneuos biliary drainage, hospitalized | 0 | 0 | 0 | 0 | 1 |  |
| **Pre-defined patient groups: Specified infections** | |  |  |  |  |  |  |
| Brandtzaeg et al 1989 [11] ^a^ | Meningococcal disease, hospitalized |  |  |  |  | 24 | 11 |
| Brandtzaeg et al 1996 [12] ^a^ | Meningococcal disease, hospitalized |  |  |  |  | 40 | 19 |
| Butler et al 1973 [13] ^b^ | Plague, hospitalized |  |  |  |  | 2 | ND |
| Butler et al 1976 [14] ^b^ | Plague, hospitalized |  |  |  |  | 3 | 2 |
| Magliulo et al 1976 [15] ^c^ | Salmonellosis, hospitalized |  |  |  |  | 1 | ND |
| Magliulo et al 1976 [15] ^c, d^ | Typhoid, hospitalized |  |  |  |  | 8 | 4 |
| Adinolfi et al 1987 [16] ^d^ | Typhoid, hospitalized |  |  |  |  | 7 | 7 |
| Suyasa et al 1995 [17] | Typhoid, hospitalized |  |  |  |  | 4 | 6 |
|  |  |  |  |  |  |  |  |

**Additional material Table S1: GN bacteremia types and distributions** (continued)

|  |  |  | | | | | |
| --- | --- | --- | --- | --- | --- | --- | --- |
| Study [reference] | Patient population & location | E coli | | Pseudomonas | | All | |
|  |  | Group 1 | Group 2 | Group 1 | Group 2 | Group 1 | Group 2 |
|  |  | Etx+ | Etx- | Etx+ | Etx- | Etx+ | Etx- |
|  |  |  |  |  |  |  |  |
| **Pre-defined patient groups: Oncology and transplant patients** | |  |  |  |  |  |  |
| Bion et al 1994 [18] | Elective liver transplantation (100% immunosupressed), ICU | 0 | 0 | 1 | 1 | 1 | 1 |
| Engervall et al 1997 [19] ^a^ | Febrile, oncology (80% neutropenic), hospitalized | 1 | 0 | 1 | 1 | 2 | 4 |
| Hynninen et al 1995 [20] | Febrile, oncology (43% neutropenic), hospitalized | 1 | 11 | 0 | 1 | 3 | 24 |
| Yoshida et al 1994 [21] ^a^ | Febrile, oncology (63% neutropenic), hospitalized | 1 | 0 | 4 | 3 | 21 | 9 |
| **Pre-defined patient groups: Other** | |  |  |  |  |  |  |
| Byl et al 2001 [22] ^a^ | Suspected sepsis, hospitalized | 6 | 2 | 0 | 0 | 8 | 4 |
| Giamarellos et al 1999 [23] ^a^ | Acute pyelonephritis, hospitalized | 2 | 8 | 0 | 0 | 3 | 9 |
| Levin et al 1972 [24] | Suspected sepsis, hospitalized | 4 | 3 | 6 | 1 | 20 | 14 |
| Stumacher et al 1973 [25] | Suspected bacteremia, hospitalized | 7 | 11 | 2 | 4 | 28 | 37 |
| **Adult patients in ICU with sepsis as contemporaneously defined** | |  |  |  |  |  |  |
| Bates et al 1998 [27] ^a^ | Sepsis syndrome, multi-center cohort, hospitalized | 3 | 20 | 2 | 3 | 10 | 39 |
| Danner et al 1991 [29] ^a^ | Clinically defined septic shock, ICU | 5 | 4 | 2 | 0 | 11 | 8 |
| Dofferhoff et al 1992 [30] | Clinically defined severe sepsis, ICU | 1 | 1 | 2 | 0 | 4 | 2 |
| Goldie et al 1995 [31] ^a^ | Sepsis syndrome, ICU | 1 | 1 | 2 | 0 | 9 | 3 |
| Guidet et al 1994 [32] ^a^ | Sepsis syndrome, ICU | 9 | 7 | 0 | 0 | 24 | 9 |
| Strutz et al 1999 [33] | Sepsis syndrome, ICU | 4 | 4 | 0 | 1 | 5 | 5 |
| Opal et al [35]  (Low; >20 pg/ml) ^a, e^ | Sepsis syndrome, multi-center cohort, ICU | 15 | 6 | 8 | 7 | 51 | 21 |
| Opal et al [35]  (High; >660 pg/ml) ^a, e^ | Sepsis syndrome, multi-center cohort, ICU | 18 |  | 16 |  | 63 |  |

**Additional material Table S1: GN bacteremia types and distributions (continued) (continued)**

Etx, Endotoxemia; GNB, Gram negative bacteremia; ND, no data; ICU, intensive care unit.

1. Data for these studies [7, 9, 11, 12, 19, 21-23, 27, 29, 31, 32, 35] provided by personal communication.
2. Two studies of Plague were aggregated for this analysis [13, 14].
3. This study stratified into two sub-studies of Typhoid (adults) and Salmonellosis (pediatric) [15].
4. Two studies of Typhoid were aggregated for this analysis [15, 16].
5. The mortality proportion data for patients with endotoxemia detected from this study [35] has been stratified at two breakpoints
